# Antibiotic-Resistant Pyrexia of Unknown Origin: An Unusual Presentation of Myelodysplastic Syndrome

**DOI:** 10.7759/cureus.15739

**Published:** 2021-06-18

**Authors:** Abubakar Tauseef, Avdesh Buragadda, Sunil Nair, Ayesha Anum, Maryam Zafar

**Affiliations:** 1 Internal Medicine, Dow University of Health Sciences, Karachi, PAK; 2 Internal Medicine, Creighton University School of Medicine, Omaha, USA

**Keywords:** antibiotic, pyrexia of unknown origin, review, myelodysplastic syndrome, mystery

## Abstract

A 74-year-old male presented to the emergency department (ED) with complaints of high-grade fever, productive cough, and difficulty breathing for three weeks, and a significant weight loss in the past six months. Despite a detailed investigation, diagnosis could not be achieved. Multiple empiric regimens were tried but no response was observed. Bone marrow biopsy was conducted after suspicious peripheral film result, which concluded that the patient was having myelodysplastic syndrome. Although pyrexia of unknown origin has a diverse group of diseases to evaluate for, and rare entities are difficult to diagnose, meticulous evaluation may give significant evidence for targeted diagnostic workup.

## Introduction

The term “pyrexia of unknown origin” (PUO) was first described by Petersdorf and Beeson in 1961, with three postulated criteria, which include a fever of greater than 38.3°C on multiple occasions and for more than three weeks and investigations yielding no diagnosis after one week of hospital admission [1,2]. The etiology behind this clinical quandary has been classified into four major categories: (1) infections, (2) inflammatory conditions, (3) neoplasia, and (4) miscellaneous [3]. Although rare, several conditions with compromised hematological functions may present initially as PUO [3]. Yet, myelodysplasia as a cause behind has been rarely reported in the literature [4]. This case report is an effort to establish the understanding of an unusual presentation of myelodysplastic syndrome (MDS) as antibiotic-resistant PUO.

## Case presentation

A 74-year-old male of African American ethnicity with a past medical history of chronic obstructive lung disease (on room air at home), smoking, alcohol use, and no history for exposure to the cytotoxic agent but a significant family history of sarcomas and lymphomas in the paternal family presented to the Emergency Department (ED) with a fever of 103.1°F, productive cough, progressive shortness of breath, and dyspnea on exertion happening for the last three weeks. He also reported a significant weight loss of 20 pounds in the last six months. He never traveled outside the United States, with no prior exposure or close contact with tuberculosis patients. He had a significant history of admission to the hospital due to pneumonia-induced respiratory failure during which he was intubated and sedated. He denied headache, altered level or loss of consciousness, abdominal pain, nausea, vomiting, diarrhea, dysuria, or any recent flu-like illness.

Physical examination was unremarkable except a temperature of 103.0°F, pulse of 123 beats/minute, blood pressure of 100/70 mmHg, respiratory rate of 32 per minute, generalized pallor, petechiae over the trunk and bilateral lower limb, and crackles in all lung fields in bilateral lungs. Laboratory investigation showed a hemoglobin level of 5.4 gram/dL, white blood cell count of 0.8 cells/mm^3^ with an absolute neutrophil count of 0.2 cells/uL, platelet count of 18,000 cells/mm^3^, procalcitonin of 15 ng/mL, lactate dehydrogenase of 5.7 U/L, erythrocyte sedimentation rate of 35 mm/hour, and reticulocyte count of 1.5%; complete metabolic panel and liver function test were within normal limits. Anti-nuclear antibodies (ANA) were negative, direct or indirect Coombs test was within normal limit, urine analysis was unremarkable, and blood, urine, and sputum culture showed no growth. CT scan of paranasal sinuses, chest, abdomen, and pelvis with or without contrast was unremarkable. Initial differential after getting blood work included drug-induced aplastic anemia, myelodysplasia, PUO, and nutritional deficiency

The patient received a couple of blood transfusions, was transferred to the isolation floor, blood, urine, and sputum cultures collected, and was started on intravenous vancomycin and piperacillin/tazobactam with filgrastim on day 1 of the admission as a broad-spectrum antibiotic for fever and to improve marrow response, but he never responded to the medications. Bone marrow biopsy was sent as suspicion of aplastic anemia or MDS was made, and the patient was tested for hepatitis panel, HIV, pneumococcal antigen, legionella, 1-3 B-D-glucan, mycobacterium tuberculosis, histoplasmosis, coccidioidomycosis, and Q-fever to determine the cause of fever as per infectious disease department recommendations. On day 14 of hospitalization, the infectious disease department was consulted, and they started the patient on voriconazole, azithromycin, and meropenem instead of vancomycin and piperacillin/tazobactam. All the antigen or serology tests were unremarkable. Bronchoalveolar lavage was sent for culture sensitivity for fungal infections, which came out to be negative. The patient was continuously running a fever of 101-102°F despite the use of multiple broad-spectrum antibiotics as per infectious disease department recommendations. Reviewing peripheral smear of blood revealed 10% blast cells and 23% segments and bands. A bone marrow biopsy was conducted, which showed myelodysplasia with an excess blast of 13% and hypercellular marrow (80%), and flow cytometry showed positivity for CD34 positive myeloblast and CD117 erythroblast ruling in MDS, but evidence of acute myeloid leukemia (AML) was ruled out. A family meeting was called to discuss further management options with the patient and family. After the discussion with the family, the patient signed the do not resuscitate (DNR) form and went for palliative care as no medication was helping him feel better.

## Discussion

Since it was first labeled as an entity, PUO has been a question of a physician’s expertise to figure out the inciting pathology among a widespread category behind this clinical entity. Moreover, inadequate marrow response contributes to many cases of PUO [4]. Ben-Baruch et al. studied 75 patients with PUO and discovered that 16 were associated with some hematological disease; still, among those diseases that affect hematological mechanism and presents as PUO, MDS was studied to be one out of 16, suggesting MDS as being a rare entity to manifest as PUO [4].

MDS represents the premalignant condition of ineffective hematopoiesis with marrow showing dysplastic changes and peripheral film showing cytopenia of one or more cell lines [5]. A significant clue can be driven by the abnormal peripheral smear during basic workup showing abnormal or immature cells to estimate the hematological abnormality [3]. As in our patient, when despite an extensive workup to evaluate the underlying mechanism behind PUO, the diagnosis remained unclear, and various empiric therapies could not help control the deteriorating condition of the patient, a peripheral film leads to the evaluation of compromised hematological response in an otherwise infective process. A bone marrow biopsy was performed afterward, which clearly showed increased cellularity of the marrow and the presence of blast cells. The usual diagnostic criteria for MDS involve peripheral film, bone marrow aspirate, and biopsy. Moreover, cytometry, cytogenetics, and molecular genetics support the diagnosis and aid in further classification of MDS [6]. Although our patient’s cytogenetics was inconclusive, peripheral and bone marrow showed the clues for diagnosis.

A similar case was reported by Cunha et al., where the patient was initially diagnosed with still’s disease while managing his PUO but the persistent monocytes lead evidence towards further investigation, and bone marrow biopsy confirmed MDS as the actual pathology [7]. Another case described an old-aged female who presented with PUO and pancytopenia and was initially diagnosed with brucella, and during treatment the actual cause of her symptoms was found to be due to MDS [8].

Towards the prognosis evaluations, blast cell percentage in bone marrow gives significant predictions for the disease course [9], where the risk of conversion to AML is directly proportional to the number of blast cells in the marrow and >20 % blast cells are categorized as AML [10]. Our patient had 13% of blast cells in his marrow, ruling out the transition of MDS to AML. Furthermore, studies support the association of cytotoxic drugs and the development of myelodysplasia, but there is a paucity of literature about the genetic predisposition of myelodysplasia in a person with a family history of sarcoma and lymphoma [11], as in our patient.

## Conclusions

PUO has been a challenge since its inception, although recent advances in diagnostic tools help clinicians narrow down the potential causes. Even now some rare entities are frequently overlooked. The purpose of this report is to share an uncommon underlying MDS behind pyrexia's unknown origin. Therefore, it is highly recommended to explore hematopoiesis while dealing with such cases.
